# Cone Beam Computed Tomography Panoramic Mandibular Indices in the Screening of Postmenopausal Women with Low Bone Mass: Correlations with Bone Quantity and Quality

**DOI:** 10.3390/dj12080256

**Published:** 2024-08-14

**Authors:** Ioana Ruxandra Poiană, Iulia Florentina Burcea, Silviu-Mirel Pițuru, Alexandru Bucur

**Affiliations:** 1 Faculty of Dentistry, “Carol Davila” University of Medicine and Pharmacy, 050474 Bucharest, Romania; ioana.poiana@drd.umfcd.ro (I.R.P.); silviu.pituru@umfcd.ro (S.-M.P.); alexandru.bucur@umfcd.ro (A.B.); 2 Faculty of Medicine, “Carol Davila” University of Medicine and Pharmacy, 050474 Bucharest, Romania; 3 Department of Endocrinology, National Institute of Endocrinology C. I. Parhon, 011853 Bucharest, Romania

**Keywords:** osteoporosis, menopause, cone beam computed tomography

## Abstract

Objective. This study examined the potential use of computed tomography panoramic mandibular indices on cone beam CT (CBCT) for assessing bone density in postmenopausal women with low bone mass. Study design. The study enrolled 104 postmenopausal women who underwent dual-energy X-ray absorptiometry (DXA) using a DXA scanner and mental foramen region CBCT alongside the NewTom VGi EVO Cone Beam 3D system. We assessed the relationship between the following DXA parameters: lumbar, femoral neck, and total hip T score, bone mineral density (BMD), and lumbar trabecular bone score (TBS). The following panoramic mandibular indices were also considered: the computed tomography mandibular index superior (CTI(S)), computed tomography mandibular index inferior (CTI(I)), and computed tomography mental index (CTMI). Results. The study revealed moderate correlations between CBCT indices and BMD/TBS scores: CTMI showed the highest correlation with the femoral neck T-score (r = 0.551, *p* < 0.0001). TBS scores were also moderately correlated with CBCT indices: CTMI showed a moderate positive correlation with TBS (r = 0.431, *p* < 0.0001); CTI(S) had a similar moderate positive correlation with TBS (r = 0.421, *p* < 0.0001). AUC values ranged from 0.697 to 0.733 for osteoporosis versus the osteopenia/normal group and from 0.734 to 0.744 for low versus normal bone quality groups, *p* < 0.0001. The comparison of the values of the studied indices between low versus normal bone quality (quantified with TBS) groups showed high sensitivity but low specificity. Conclusions. CBCT-measured indices CTI(S), CTI(I), and CTMI are useful in assessing patients with low bone mass to improve, by specific treatment, the prognosis of dental implants.

## 1. Introduction

First defined in 1993, osteoporosis is a widespread bone condition marked by reduced bone density and the structural weakening of bone tissue, leading to heightened bone fragility and an increased risk of fractures [1,2]. Although this condition can affect almost any person from any age group, postmenopausal women are a population “at-risk” of suffering from osteoporosis, mainly because of the changes in estrogen levels [3]. For the measurement of bone mineral density (BMD), dual-energy-X-ray absorptiometry (DXA) is the gold standard for the diagnosis of osteoporosis [4] and is defined as follows, according to the American Association Of Clinical Endocrinologists and American College Of Endocrinology (AACE/ACE) Clinical Practice Guidelines criteria: a. T-score –2.5 or below in the lumbar spine, femoral neck, total, and/or 33% (one-third) radius; b. low-trauma spine or hip fracture (regardless of BMD); c. osteopenia or low bone mass (T-score between −1 and −2.5) with fragility fracture of the proximal humerus, pelvis, or possibly distal forearm; and/or d. low bone mass or osteopenia and high FRAX^®^ fracture probability based on country-specific thresholds [5].

The threshold for diagnosing osteoporosis based on BMD is important for clinical assessment and treatment, but although it is the best tool to predict fracture risk, it has several limitations that reduce its effectiveness in identifying patients who will eventually experience a fracture [1,6].

Knowing that there might be a significant correlation between the BMD of the mandible and that in the femoral neck and lumbar spine measured in patients with osteoporosis [7,8], some tools like cone beam computed tomography (CBCT), which assesses jaw bone density and quality might be used to evaluate BMD in patients with low bone mass [9,10]. Barra et al. [9] stated that radiomorphometric indices measured in specific centers from mental foramina are promising tools in CBCT of the mandible for assessing the BMD postmenopause, exhibiting significant differences among patients with normal BMD and the ones with osteopenia and/or osteoporosis. Similarly, Koh et al. [8] reported that specific indices on CBCT axial, sagittal, and coronal images are useful methods for osteoporosis screening. These panoramic quantitative indices are the most used method to evaluate low bone mass in the mental foramina region. By using superior and inferior cortical indices (CTI(S) and CTI(I)), significant differences were found between women with normal bone mass and those with osteoporosis (*p* < 0.05). The present study highlighted the effectiveness of these CBCT-derived indices in distinguishing between varying levels of bone mass and assessing the above-mentioned mandibular CBCT indices in primary osteoporosis with regard to both quantity and quality.

The evaluation of bone mass before dental implants is valuable for prognosis because the identification of patients with low bone mass and performing specific treatment can increase implant survival and stability.

## 2. Materials and Methods

This study was performed on postmenopausal women. Written informed consent was obtained from patients before the study. The study was approved by the Ethics Committee of “C. I. Parhon” National Institute of Endocrinology, Bucharest, Romania.

The inclusion criteria were as follows: female sex; aged more than 50 years; DXA with TBS evaluation; biological evaluation; and CBCT evaluation. The exclusion criteria were as follows: the presence of systemic diseases affecting bone metabolisms, such as neoplasia, osteomalacia/history of rickets, endocrine disorders (hyperthyroidism, hyperparathyroidism, Cushing’s syndrome, or acromegaly), severe renal failure, liver failure, or malabsorption disorders (Crohn disease, celiac disease); the presence of rheumatologic diseases (rheumatoid arthritis, ankylosing spondylitis), a history of oophorectomy; and the use of medications interfering with bone turnover/density (glucocorticoids, aromatase inhibitors, selective serotonin reuptake inhibitors, medroxyprogesterone acetate, antiepileptic drugs, unfractionated heparin).

The patients included were candidates for dental implants, and they performed CBCT as part of the pre-implant protocol, with the CBCT evaluation being commonly used in most of the dental clinics in a personalized implant protocol. The patients were evaluated in close collaboration with a private provider of dental imaging with expertise in CBCT imaging and the National Institute of Endocrinology, which is a public hospital that attends hundreds of patients with metabolic bone disease yearly. The enrolment period was between April 2021 and April 2024, with a maximum distance of 2 months between CBCT and DXA evaluations.

The included postmenopausal women had normal BMD, osteopenia, or osteoporosis, with or without antiresorptive/anabolic treatment.

Written informed consent was obtained from the patients before the study. The study was approved by the Ethics Committee of “C. I. Parhon” National Institute of Endocrinology, Bucharest, Romania (no. 4/8 April 2021).

### 2.1. CBCT Measurements

According to the modified Ledgerton’s classification [11], we used the following panoramic mandibular indices: CTI(S), CTI(I), and CTMI, defined as follows:

- CTI(S): superior CT mandibular index (the ratio of the inferior cortical width to the distance from the superior margin of the mental foramen to the inferior mandibular border);
- CTI(I): inferior CT mandibular index (the ratio of the inferior cortical width to the distance from the inferior margin of the mental foramen to the inferior mandibular border);
- CTMI: CT mental index (the inferior mandibular cortical width).

The measurements of S, I, and W indices were performed on coronal images. The W index represents the inferior cortical width of the mandible; the S index represents the distance from the superior margin of the mental foramen to the inferior mandibular border; the I index represents the distance from the inferior margin of the mental foramen to the inferior mandibular border (Table 1). The measurements were performed by an experienced radiologist.

The CBCT images were obtained using NewTom VGi EVO Cone Beam 3D Imaging (CEFLA s.c.—Via Selice Provinciale 23/a, Imola, Italy) at 110 kV, 7.5 mA, 3.5 s, with a pixel size 0.2 mm. The images were reconstructed using NewTom NNT (ISDP©10003:2020 compliant in accordance with EN ISO/IEC 17065:2012 certificate number 2019003109-2) with Viewer software, version 16.0.

### 2.2. Bone Mineral Density Measurements

BMD was measured at the lumbar spine (LS), femoral neck (FN), and total hip by DXA (General Electric Prodigy Lunar, Bedford, UK) using enCore Software 10,50,086. BMD was expressed in grams per square centimeter (g/cm^2^), and, by comparing the BMD with the peak bone mass of a young adult, a T-score was obtained, expressed in standard deviations (SDs) and a Z-score for age-matched SDs. All measurements were performed according to the International Society for Clinical Densitometry (ISCD) [12]

TBS values were obtained by analyzing the L1–L4 vertebrae DXA images with iNsight Software version 2.2.0.0 (Medimaps Group SA Headquarters, Geneva, Switzerland).

All patients were scanned on the same DXA machine, yet by two different operators, thus allowing a user bias.

## 3. Statistical Analysis

We statistically analyzed the patients based on the value of the BMD (lumbar spine, femoral neck, total hip scores) and T score (lumbar spine, femoral neck, total hip scores), respectively, and TBS as continuous values, regardless of the osteoporosis diagnosis at the time of the CBCT evaluation.

Alternatively, we used binary logistic analysis to divide the patients based on osteoporosis diagnosis (according to AACE/ACE criteria) [5]. We also employed the regression analysis, the t-test, Pearson’s correlation coefficient, and Spearman’s rho, using IBM SPSS Statistics, version 25 (SPSS Inc, Chicago, IL, USA) for Mac OS.

## 4. Results

The characteristics of the patients included in the study are presented in the table below (Table 2). We included 104 postmenopausal women in this study with a mean age of 65.15 ± 9.12 years old. The mean age for menopause was 47.22 ± 5.3 years old, with a mean body mass index (BMI) of 26.58 ± 7.48. The mean lumbar BMD score was 0.944 g/cm^2^ (0.544, 1.437) with a mean T lumbar score of −1.95 SD (−5.3, 2.1). For the femoral neck, the mean BMD score was 0.944 g/cm^2^ (0.554, 1.221) with a mean T score of −1.95 SD (−3.5, 1.3). The mean score of TBS was 1.284 g/cm^2^ ± 105.83 SD (1.062, 1.558).

The patients had significantly lower T scores and BMD values in the osteoporosis group compared to the normal/osteopenia group.

Table 3 lists the mean values of the CBCT parameters depending on the bone quantity (assessed with lumbar and femoral neck T-scores) and bone quality (assessed with TBS). There were statistically significant differences based on bone quality and quantity, with higher values of the studied CBCT parameters observed in patients with a higher T score and higher TBS (*p* < 0.0001). Postmenopausal women with normal bone quality also show higher CTMI values (see Table 3). The values of CTI(S) were higher for patients with intermediate and normal bone quality.

Statistically significant correlations were also observed between bone quantity; this time, it was expressed as the linear value using the lumbar, femoral neck, and total hip BMD, not just using the T-score from the three mentioned regions. This emphasizes the importance of BMD to assess bone quantity more than just the T-score (Table 4). All CBCT parameters were moderately correlated (with the highest correlation coefficient being 0.551 for the CTMI with a femoral neck T-score; see Table 4). Using Pearson’s correlation coefficient and Spearman’s rho, we found the same moderate correlation between CBCT indices and bone mass (Table 5).

This study revealed moderate correlations between CBCT indices and BMD/TBS scores. Specifically,

- CTMI showed the highest correlation with the femoral neck T-score (r = 0.551, *p* < 0.0001).
- CTI(S) and CTI(I) also displayed significant correlations with T-scores and BMD measurements across various sites (lumbar spine, femoral neck, and total hip), with correlation coefficients ranging from 0.322 to 0.522 (*p* < 0.0001).
- TBS scores were also moderately correlated with CBCT indices, with CTMI having a correlation coefficient of 0.431 (*p* < 0.0001).

Using regression analysis (linear and logistic), we list in Table 6 and Table 7 the prediction power of the studied CBCT parameters for assessing bone mass. We found that CTMI, CTI(I), and CTI(S) were effective in predicting bone quality and quantity (*p* < 0.0001). In the logistic regression analysis, all models reached statistical significance (see Table 7).

**Figure 1:**
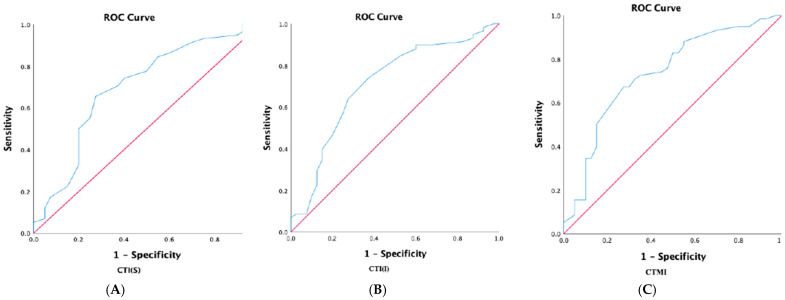
Receiver operator characteristic (ROC) analyses for the pair-wise comparisons of indices in patients with osteoporosis and osteopenia/normal BMD. (**A**)—comparison of CTI(S) index between individuals with T score −2.5 SD versus > −2.5 SD; (**B**)—comparison of CTI(I) index between individuals with T score −2.5 SD versus > −2.5 SD; and (**C**)—comparison of CTMI index between individuals with T score −2.5 SD versus > −2.5 SD; osteoporosis diagnosed based on T score values in specific sites, osteoporosis if ≤−2.5 SD and osteopenia/normal if >−2.5 SD; BMD, bone mass density; expressed as g/cm^2^; SD, standard deviation; CBCT, cone-beam computer tomography; CTMI, computer tomography mental index; CTI(I), inferior computer tomography mandibular index; and CTI(S), superior computer tomography mandibular index.

**Figure 2:**
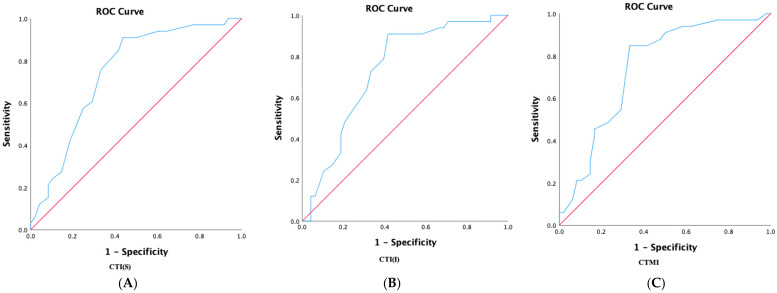
Receiver operator characteristic (ROC) analyses for the pair-wise comparisons of indices in patients with low versus normal bone quality based on TBS. (**A**)—comparison of CTI(S) index between individuals with low versus normal bone quality, based on TBS; (**B**)—comparison of CTI(I) index between individuals with low versus normal bone quality based on TBS; and (**C**)—comparison of CTMI index between individuals low versus normal bone quality, based on TBS; trabecular bone score, expressed as low if TBS ≤ 1.31 g/cm^2^, and normal if TBS > 1.31 g/cm^2^; TBS, trabecular bone score; SD, standard deviation; CBCT, cone-beam computer tomography; CTMI, computer tomography mental index; CTI(I), inferior computer tomography mandibular index; and CTI(S), superior computer tomography mandibular index.

ROC analyses for the pair-wise comparisons between the groups are illustrated in Figure 1 and Figure 2. The AUC values, 95% CI, sensitivity, specificity, and cut-off points of cortical thickness are listed in Table 6. The comparison between individuals based on the T score value (lumbar, and/or femoral neck and/or total hip) with osteoporosis (T score ≤ −2.5 SD) and osteopenia/normal value (T-score > −2.5 SD) yielded an AUC between 0.697 and 0.733 (see Figure 1). The cut-offs are presented in Table 8, with a sensitivity between 63.8% and 67.2% and a specificity of 27.5%.

The comparison between individuals based on TBS (expressed as low if TBS ≤1.31 g/cm^2^ and normal if TBS > 1.31 g/cm^2^) yielded an AUC between 0.734 and 0.744 (see Figure 2). The cut-offs are presented in Table 8, with a sensitivity between 84.8% and 90.9% and a specificity between 33.3% and 43.8%.

## 5. Discussion

This study examined the use of computed tomography panoramic mandibular indices on CBCT images for the evaluation of BMD in postmenopausal women. The results demonstrated that CTMI, CTI(S), and CTI(I) are tools that can be used in mandibular CBCT for the assessment of bone mass in postmenopausal women; these indices exhibit significant differences among this category of patients depending on the bone mass quantity and quality evaluated on DXA scan.

The lowest T score observed in this study is for the lumbar spine in the context of the rich trabecular bone content in this area, which is more susceptible to bone changes [13]. The use of all validated DXA sites and comparing patients with normal/osteopenia/osteoporosis status rather than comparing only osteoporosis patients with normal bone mass [9,14] enhances the depth of the present results. Assessing bone quality through the trabecular bone score offers insights into bone micro-architecture beyond just bone quantity, making it a valuable tool for evaluating bone health in cases of both primary and secondary osteoporosis.

The study found significant correlations between the panoramic mandibular indices and lumbar T-scores. The computed tomography mental index (CTMI), computed tomography mandibular index superior (CTI(S)), and computed tomography mandibular index inferior (CTI(I)) showed the following Pearson correlation coefficients with lumbar T-scores as follows: CTMI: r = 0.429, *p* < 0.0001; CTI(S): r = 0.387, *p* < 0.0001; CTI(I): r = 0.364, *p* < 0.0001. These results indicate a moderate positive correlation, suggesting that as the lumbar T-score increases, indicating higher bone density, the values of the mandibular indices also tend to increase. The correlations with femoral neck T-scores were even stronger. The higher correlation coefficients reflect a stronger relationship between mandibular indices and femoral neck bone density compared to the lumbar spine. Similarly, total hip T-scores also showed significant correlations. These findings align with the scores from Koh and colleagues [8,15], who reported that these CBCT indices could be effective for osteoporosis screening and the evaluation of bone quality in postmenopausal women.

The findings of the study suggest that mandibular indices might be particularly sensitive to changes in the bone density of the femoral neck, considering that the highest correlation was observed between CTMI and femoral neck T-scores. In a study by Munhoz et al. [16], significant correlations were found between mandibular cortical width and BMD. This study corroborates the present findings, particularly regarding the strong correlation between CTMI and femoral neck BMD, reinforcing the potential of mandibular indices as indicators of overall bone health. Barra et al. [17] also found significant differences in mandibular radiomorphometric indices among patients with normal BMD or a low bone mass. Their results on 48 postmenopausal women emphasized the potential of CBCT measurements to reflect systemic bone density changes.

TBS is a relatively new metric derived from lumbar DXA scans. Unlike BMD, which measures bone mineral density, TBS provides an indirect assessment of the trabecular micro-architecture. It is a valuable tool in evaluating bone micro-architecture and predicting fracture risk as it reflects the structural integrity of trabecular bone, which is crucial in understanding bone strength and fragility. The current study explored the correlation between CBCT-derived mandibular indices and TBS. The key findings are as follows: CTMI showed a moderate positive correlation with TBS (r = 0.431, *p* < 0.0001); CTI(S) had a similar moderate positive correlation with TBS (r = 0.421, *p* < 0.0001); CTI(I) also correlated with TBS, albeit slightly lower than the other two indices (r = 0.351, *p* < 0.0001). These correlations indicate that higher mandibular index values, as measured by CBCT, are associated with better trabecular bone quality, as indicated by TBS scores. Diba SF et al. [18] stated that the depletion of the trabeculae bone structure is much clearer in osteoporotic postmenopausal women; thus, the initial bone quality assessment can be performed using trabecular thickness parameters. However, in their study, they used dental radiographs as the bone quality screening tool. This is the first study to correlate TBS with panoramic mandibular CBCT indices regarding the assessment of bone micro-architecture (a parameter of bone quality) in postmenopausal women.

The comparison of the studied indices between the low and normal bone quality groups (quantified with TBS) demonstrated high sensitivity with a low chance of false-negative results. The low specificity indicates a high probability of false positive results. In contrast, the comparison of mean values of the indices between osteoporosis and normal BMD/osteopenia groups revealed lower sensitivity, suggesting a higher possibility of false-negative results.

This study highlights some of the limitations of DXA, including its ineffectiveness in predicting fractures accurately. Although it remains the gold standard in the diagnosis of osteoporosis [19], the published literature [12,20] has noted that a significant limitation of BMD measurements is that most fragility fractures occur at non-osteoporotic BMD levels (T-scores > −2.5), which undermines its effectiveness as a screening tool [12]. A prospective study from 2024 performed by Sornay-Rendu et al. [21] used high-resolution peripheral computed tomography (HR-pQCT) to evaluate the micro-architecture of the radius in almost 600 postmenopausal women. It found that even patients with a normal BMD who experience fragility fractures have altered bone micro-architecture. In a study from the same year by Li et al. [13], who also used QCT to evaluate the predictive value of thoracic trabecular BMD, it was found that this correlated with the risk of future hip and vertebral fractures, with most of the patients exhibiting underdiagnosed low bone mass (osteopenia/osteoporosis).

Another imaging exam that can be useful for the maxillofacial region is magnetic resonance imaging (MRI) [22]. While CBCT is the standard imaging for the maxillofacial bone area, MRI can provide comparable results [23], although it can slightly overestimate bone parameters [24]. Some studies have reported similar bone results when using MRI and other imaging techniques such as CT and CBCT [23]. In addition, one study found a weak correlation between MRI and CBCT scan values [24]. The differences between MRI and CBCT are measurable concerning spatial resolution or image contrast, thus affecting the detection of discrete changes in bone morphology or trabecular arrangement [22].

Our findings suggest that recognizing the potential role of CBCT in assessing bone mass could enhance the early detection and management of osteoporosis in postmenopausal women. Thus, the patients can be treated in order to increase bone mass and improve bone micro-architecture, enabling the clinician to provide a more precise, patient-specific prognosis for future implant success rather than relying on the typical summary evaluation. Since CBCT can be more accessible and less cumbersome than DXA, it offers a practical approach for widespread osteoporosis screening in addition to existing tools, especially when considering the increased use of this tool in the general population. This is particularly important given the increasing prevalence of osteoporosis among aging populations.

## 6. Conclusions

The findings of this study suggest that CBCT-derived mandibular indices can be reliable indicators of low bone mass in postmenopausal women. These indices could potentially be integrated into routine dental assessments, providing an accessible screening tool for osteoporosis. This is important to evaluate bone mass before the dental implant and treatment accordingly to obtain a stable implant site and increase its prognosis.

Future research should aim to validate these findings in larger, more diverse populations, explore the longitudinal predictive value of these indices for fracture risk, and investigate the cost-effectiveness and practical implementation of CBCT screenings in clinical practice.

## Figures and Tables

**Table 1:** Indices and their significance.

Index	Value
CTI(S)	W/S
CTI(I)	W/I
CTMI	W

W—inferior cortical width of the mandible; S—distance from the superior margin of the mental foramen to the inferior mandibular border; and I—distance from the inferior margin of the mental foramen to the inferior mandibular border.

**Table 2:** Patients’ distribution.

Parameter	T score ≤ −2.5 *	T score ≥ −2.5 *
Number	45	59
BMI (kg/m^2^)	24.47 ± 4.91	27.66 ± 8.38
Age at menopause (years)	47.18 ± 4.49	47.43 ± 5.73
Femoral neck T score (SD)	−2.06 ± 0.66	−1.15 ± 0.85
Total hip T score (SD)	−1.67 ± 0.78	−0.6 ± 1.04
Lumbar T score (L1-L4) (SD)	−3.12 ± 0.69	−1.3 ± 0.92
Femoral neck BMD (g/cm^2^)	0.727 ± 0.082	0.852 ± 0.11
Total hip BMD (L1-L4) (g/cm^2^)	0.713 ± 0.366	0.941 ± 0.132
Lumbar BMD (g/cm^2^)	0.805 ± 0.088	1.018 ± 0.012
TBS score (g/cm^2^)	1207 ± 181	1313 ± 100.9

* The values are expressed as the mean ± SD but did not include patients with osteoporosis based on other AACE criteria.

**Table 3:** Mean values of the computed tomography parameters on cone beam computed tomography (CBCT) images.

CBCT Parameter	Lumbar T-Score > −2.5 SD (*n* = 62)	Lumbar T-Score ≤ −2.5 SD (*n* = 42)	Femoral Neck T-Score > −2.5 SD (*n* = 85)	Femoral Neck T-Score ≤ −2.5 SD (*n* = 14)	Low Bone Quality (TBS ≤ 1.23, *n* = 21)	Intermediate Bone Quality (TBS > 1.23 and <1.31, *n* = 32)	Normal Bone Quality (TBS ≥ 1.31, *n* = 33)	*p* Value
CTMI	2.74 ± 0.80	2.43 ± 0.83	2.72 ± 0.74	1.84 ± 0.68	2.12 ± 0.61	2.42 ± 0.84	3.04 ± 0.69	*p* < 0.0001
CTI(I)	0.24 ± 0.07	0.21 ± 0.07	0.24 ± 0.06	0.17 ± 0.06	0.20 ± 0.06	0.21 ± 0.07	0.26 ± 0.05	*p* < 0.0001
CTI(S)	0.18 ± 0.05	0.16 ± 0.05	0.18 ± 0.05	0.13 ± 0.06	0.14 ± 0.04	0.16 ± 0.05	0.20 ± 0.04	*p* < 0.0001

TBS, trabecular bone score; SD, standard deviation; CBCT, cone-beam computer tomography; CTMI, computer tomography mental index; CTI(I), inferior computer tomography mandibular index; and CTI(S), superior computer tomography mandibular index.

**Table 4:** Correlations between CBCT parameters and bone quantity and quality parameters.

Parameters for Correlations ^†^	CTMI	CTI(S)	CTI(I)
Lumbar T score *	0.429, *p* < 0.0001	0.387, *p* < 0.0001	0.364, *p* < 0.0001
Femoral neck T score *	0.551, *p* < 0.0001	0.465, *p* < 0.0001	0.481, *p* < 0.0001
Total hip T score *	0.470, *p* < 0.0001	0.440, *p* < 0.0001	0.451, *p* < 0.0001
TBS score *	0.431, *p* < 0.0001	0.421, *p* < 0.0001	0.351, *p* < 0.0001
TBS quality assessment ***	0.454, *p* < 0.0001	0.379, *p* < 0.0001	0.423, *p* < 0.001
Lumbar BMD **	0.359, *p* < 0.0001	0.355, *p* < 0.0001	0.322, *p* < 0.0001
Femoral neck BMD **	0.522, *p* < 0.0001	0.443, *p* < 0.0001	0.446, *p* < 0.0001
Total hip BMD **	0.509, *p* < 0.0001	0.445, *p* < 0.0001	0.460, *p* < 0.0001
Osteoporosis defined as lumbar T-score ≤ −2.5 SD	−0.387, *p* < 0.0001	−0.296, *p* = 0.003	−0.349, *p* < 0.0001

^†^ Significant at the 0.05 level t-test (2-tailed); * expressed as standard deviations; ** bone mass density, expressed as g/cm^2^; *** trabecular bone score, expressed as low if TBS ≤1.23, intermediate if TBS > 1.23 and <1.31, and normal if TBS > 1.31; TBS, trabecular bone score; SD, standard deviation; CBCT, cone-beam computer tomography; CTMI, computed tomography mental index; CTI(I), inferior computer tomography mandibular index; and CTI(S), superior computer tomography mandibular index.

**Table 5:** CBCT parameters, bone quantity, and quality parameters using Pearson’s correlation coefficient and Spearman’s rho.

Pearson’s Correlation ^†^	CTMI, (*n* = 104)	CTI(S), (*n* = 104)	CTI(I), (*n* = 103)
Lumbar T score *	0.429, *p* < 0.0001	0.387, *p* < 0.0001	0.364, *p* < 0.0001
Femoral neck T score *	0.551, *p* < 0.0001	0.465, *p* < 0.0001	0.481, *p* < 0.0001
Total hip T score *	0.470, *p* < 0.0001	0.440, *p* < 0.0001	0.451, *p* < 0.0001
TBS score *	0.431, *p* < 0.0001	0.421, *p* < 0.0001	0.351, *p* < 0.001
TBS quality assessment ***	0.454, *p* < 0.0001	0.423, *p* < 0.0001	0.379 *p* < 0.0001
Lumbar BMD **	0.359, *p* < 0.0001	0.355, *p* < 0.0001	0.322, *p* < 0.001
Femoral neck BMD **	0.522, *p* < 0.0001	0.443, *p* < 0.0001	0.523, *p* < 0.0001
Total hip BMD **	0.509, *p* < 0.0001	0.445, *p* < 0.0001	0.481, *p* < 0.0001
Osteoporosis defined as lumbar T score ≤ −2.5 SD	−0.185, *p* = 0.06	−0.176, *p* = 0.073	−0.675, *p* < 0.0001
Osteoporosis defined as femoral neck T score ≤ −2.5 SD	−0.387, *p* < 0.0001	−0.296, *p* < 0.0001	−0.349, *p* < 0.0001
Spearman’s rho	CTMI	CTI-S	CTI-I
Lumbar T score *	0.439, *p* < 0.0001	0.386, *p* < 0.0001	0.380, *p* < 0.0001
Femoral neck T score *	0.541, *p* < 0.0001	0.439, *p* < 0.0001	0.468, *p* < 0.0001
Total hip T score *	0.476, *p* < 0.0001	0.435, *p* < 0.0001	0.449, *p* < 0.0001
TBS score *	0.464, *p* < 0.0001	0.460, *p* < 0.0001	0.413, *p* < 0.0001
TBS quality assessment ***	0.456, *p* < 0.0001	0.448, *p* < 0.0001	0.419, *p* < 0.0001
Lumbar BMD **	0.397, *p* < 0.0001	0.371, *p* < 0.0001	0.363, *p* < 0.0001
Femoral neck BMD **	0.493, *p* < 0.0001	0.393, *p* < 0.0001	0.416, *p* < 0.0001
Total hip BMD **	0.518, *p* < 0.0001	0.450, *p* < 0.0001	0.473, *p* < 0.0001
Osteoporosis defined as lumbar T score ≤ −2.5 SD	−0.224, *p* = 0.022	−0.212, *p* = 0.031	−0.225, *p* = 0.022
Osteoporosis defined as femoral neck T score ≤ −2.5 SD	−0.379, *p* < 0.0001	−0.284, *p* < 0.0001	−0.328, *p* < 0.0001

^†^ Correlation is significant at the 0.01 and 0.05 level (2-tailed). * expressed as standard deviations; ** bone mass density, expressed as g/cm^2^; *** trabecular bone score, expressed as low if TBS ≤ 1.23, intermediate if TBS > 1.23 and <1.31, and normal if TBS > 1.31; TBS, trabecular bone score; SD, standard deviation; CBCT, cone-beam computer tomography; CTMI, computer tomography mental index; CTI(I), inferior computer tomography mandibular index; and CTI(S), superior computer tomography mandibular index.

**Table 6:** Predictions of osteoporosis and bone quality using regression analysis (linear regression).

Parameters	Variable	Regression Value	Constant of the Model	Model’s Sig.
Lumbar T score *	CTMI	0.70, *p* < 0.0001	−3.76, *p* < 0.0001	R^2^ = 0.18, *p* < 0.0001
Femoral neck T score *		0.46, *p* < 0.0001	−3.26, *p* < 0.0001	R^2^ = 0.3, *p* < 0.0001
Total hip T score *		0.336, *p* < 0.0001	−2.99, *p* < 0.0001	R^2^ = 0.22, *p* < 0.0001
TBS score *		0.00033, *p* < 0.0001	−0.0167 *p* = 0.09	R^2^ = 0.18, *p* < 0.0001
Lumbar T score *	CTI(S)	1.56, *p* < 0.0001	−2.00, *p* < 0.0001	R^2^ = 0.15, *p* < 0.001
Femoral neck T score *		2.61, *p* < 0.0001	−2.09, *p* < 0.0001	R^2^ = 0.21, *p* < 0.0001
Total hip T score *		9.18, *p* < 0.0001	−2.57, *p* < 0.0001	R^2^ = 0.194, *p* < 0.0001
TBS score *		0.0021, *p* < 0.0001	−0.01, *p* = 0.113	R^2^ = 0.177, *p* < 0.0001
Lumbar T score *	CTI(I)	6.97, *p* < 0.0001	−3.52, *p* < 0.0001	R^2^ = 0.13, *p* < 0.0001
Femoral neck T score *		6.63, *p* < 0.0001	−2.99, *p* < 0.0001	R^2^ = 0.23, *p* < 0.0001
Total hip T score *		7.3, *p* < 0.0001	−2.67, *p* < 0.0001	R^2^ = 0.20, *p* < 0.0001
TBS score *		0.53, *p* = 0.001	1.163 *p* < 0.0001	R^2^ = 0.112, *p* < 0.0001

* expressed as standard deviations; bone mass density, expressed as g/cm^2^.

**Table 7:** Predictions of osteoporosis and bone quality using regression analysis (logistic regression).

Parameters	Variable	Odds Ratio	Model’s Sig.
TBS quality assessment ***	CTMI	1.137, 95% CI (1.058, 1.222)	*p* < 0.0001
Osteoporosis defined as lumbar T score ≤ −2.5 SD		0.953, 95% CI (0.906, 1.003)	*p* = 0.063
Osteoporosis defined as femoral neck T score ≤ −2.5 SD		0.834, 95% CI (0.752, 0.924)	*p* < 0.001
Osteoporosis based on AACE criteria **		0.891, 95% CI (0.837, 0.948)	*p* < 0.0001
TBS quality assessment ***	CTI(S)	1.20, 95% CI (1.081, 1.333)	*p* < 0.0001
Osteoporosis defined as lumbar T score ≤ −2.5 SD		0.934, 95% CI (0.866–1.007)	*p* = 0.076
Osteoporosis defined as femoral neck T score ≤ −2.5 SD		0.836, 95% CI (0.737, 0.949)	*p* = 0.006
Osteoporosis based on AACE criteria **		0.868, 95% CI (0.796, 0.946)	*p* < 0.001
TBS quality assessment ***	CTI(I)	1.137, 95% CI (1.051, 1.230)	*p* < 0.001
Osteoporosis defined as lumbar T score ≤ −2.5 SD		0.946, 95% CI (0.892, 1.003)	*p* = 0.063
Osteoporosis defined as femoral neck T score ≤ −2.5 SD		0.847, 95% CI (0.765, 0.938)	*p* < 0.0001
Osteoporosis based on AACE criteria **		0.906 95% CI (0.849, 0.967)	*p* < 0.0001

*** expressed as standard deviations. ** bone mass density, expressed as g/cm^2^.

**Table 8:** Assessment of the area under the curve, 95% confidence interval, sensitivity, specificity, cut-off points, and model significance.

	Area under the Curve	95% CI	Sensitivity	Specificity	Cut off Point (mm)	Model Significance
Comparison between individuals based on T score *						
CTI(S)	0.697	0.588–0.806	65.5%	27.5%	1.75	*p* < 0.001
CTI(I)	0.709	0.602–0.817	63.8%	27.5%	2.35	*p* < 0.0001
CTMI	0.733	0.629–0.836	67.2%	27.5%	2.75	*p* < 0.0001
Comparison between individuals based on TBS *						
CTI(S)	0.744	0.636–0.852	90.9%	43.8%	1.55	*p* < 0.0001
CTI(I)	0.734	0.628–0.844	90.9%	41.7%	2.15	*p* < 0.0001
CTMI	0.743	0.634–0.852	84.8%	33.3%	2.70	*p* < 0.0001

* T-score expressed as standard deviations, osteoporosis if ≤−2.5 SD, and osteopenia/normal if >−2.5 SD; bone mass density, expressed as g/cm^2^; trabecular bone score, expressed as low if TBS ≤ 1.31, and normal if TBS > 1.31. TBS, trabecular bone score; SD, standard deviation; CTMI, computer tomography mental index; CTI(I), inferior computer tomography mandibular index; CTI(S), superior computer tomography mandibular index; and CI, confidence interval.

## Data Availability

The raw data supporting the conclusions of this article will be made available by the authors on request.
